# Fast Analgesic Effect in Response Test with Topical Phenytoin Cream Correlates with Prolonged Pain Relief After Extended Use in Painful Diabetic Neuropathy

**DOI:** 10.3390/ph18020228

**Published:** 2025-02-07

**Authors:** David J. Kopsky, Alexander F. J. E. Vrancken, Ruben P. A. van Eijk, Ricardo Alvarez-Jimenez, Karolina M. Szadek, Remko Liebregts, Monique A. H. Steegers

**Affiliations:** 1 Anesthesiology and Pain Management, Amsterdam UMC, Vrije Universiteit Amsterdam, De Boelelaan 1117, 1081 HV Amsterdam, The Netherlands; r.alvarezjimenez@amsterdamumc.nl (R.A.-J.); km.szadek@amsterdamumc.nl (K.M.S.); m.steegers@amsterdamumc.nl (M.A.H.S.); 2 Institute for Neuropathic Pain, 1056 SN Amsterdam, The Netherlands; 3 Department of Neurology, Brain Centre University Medical Center Utrecht, Utrecht University, 3584 CX Utrecht, The Netherlands; 4 Biostatistics & Research Support, Julius Center for Health Sciences and Primary Care, University Medical Center Utrecht, 3584 CX Utrecht, The Netherlands

**Keywords:** phenytoin cream, painful diabetic neuropathy, topical analgesia, response test

## Abstract

**Background**: Treatment of painful diabetic neuropathy (PDN) poses several challenges due to the limited effectiveness, high incidence of side effects, and potential drug interactions of oral neuropathic pain medication. Lacking systemic side effects, topical phenytoin cream offers a promising innovative approach to addressing unmet needs in neuropathic pain treatment. In this retrospective study in patients with PDN, we evaluated the analgesic effect of topical phenytoin cream in response tests and after extended use. **Methods**: We collected data from PDN patients who, prior to prolonged use of phenytoin 10% or 20% cream, either had an open response test (ORET), a single-blind (SIBRET), or a double-blind (DOBRET) placebo-controlled response test with phenytoin cream between November 2016 and February 2023. A positive ORET was defined as pain reduction of at least two points on the 11-point numerical scale (NRS) within 30 min after phenytoin cream application. A positive SIBRET or DOBRET required an additional pain reduction of 1 NRS point in the phenytoin treated area compared to the placebo. In patients with a positive response test, we evaluated the sustained pain reduction and the proportion of patients experiencing minimum pain relief of at least 30% (MPR30: moderate pain relief) and 50% (MPR50: considerable pain relief) after the extended use of phenytoin cream. We also assessed the correlation between the response test analgesic effect and the sustained pain relief. **Results**: We identified 65 patients with PDN of whom 31 (47.7%) had a positive response test. The median pain reduction in response tests was 3.0 NRS points (IQR 2.0–4.0). Extended use (median 3.3 months, IQR 1.5–12.1]) resulted in a median pain reduction of 4.0 NRS points (IQR 3.0–5.0); 26/31 (83.9%) of patients achieved MPR30, and 21/31 (67.7%) MPR50 achieved pain relief. The response test analgesic effect correlated significantly with sustained pain relief after extended use (τ = 0.72, *p* < 0.0001). **Conclusions**: In PDN patients who had a positive phenytoin cream response test, extended use of phenytoin cream provided a significant sustained pain relief.

## 1. Introduction

Diabetes mellitus affects approximately 14% of the global population, with about one-third of those individuals experiencing painful diabetic neuropathy (PDN) [1,2,3,4]. Diabetes mellitus is associated with various complications, with PDN being a common condition [5]. Other complications include retinopathy, nephropathy, cardiomyopathy, encephalopathy, and gastroparesis [5,6,7].

The pathophysiology of PDN is multifactorial and complex. Elevated glucose levels initiate altered metabolic pathways (e.g., polyol, protein kinase C, advanced glycation end products, and hexosamine pathways), leading to oxidative stress, activation of pro-inflammatory markers (e.g., TNF-α, and NF-κB), mitochondrial dysfunction, and microvascular changes, ultimately causing nerve damage [8]. Additionally, these metabolic changes create an abnormal internal environment that promotes the overexpression and sensitization of ion channels and receptors (e.g., NaV, CaV, CHN, NMDA, and TRPV) [9]. These alterations collectively result in sensory nerve hyperexcitability, leading to the development of PDN.

PDN causes a variety of symptoms: burning, painful cold, electric shocks, tingling, pins and needles, itch, and/or allodynia [4,10]. The considerable variability of these symptoms likely contributes to the frequent underrecognition and undertreatment of patients with PDN in whom pain relief is often unsatisfactory [1,11,12,13,14]. Screening questionnaires are commonly used to distinguish neuropathic pain from other types of pain. The Douleur Neuropathique 4 questionnaire (DN4) also incorporates physical examination components, such as testing for hypoesthesia and pain provoked by brushing. It has very high sensitivity (83%) and specificity (90%) and is especially useful for the screening of PDN [15,16].

The numbers needed to treat (NNT) to have at least 30% or 50% pain reduction in patients with PDN is variable: between 4 and 10 for amitriptyline, duloxetine, pregabalin, and gabapentin [17,18]. Combining these oral neuropathic pain analgesics may enhance pain relief [19]. However, a substantial proportion of patients discontinue these analgesics due to side effects, which can also exacerbate gait and balance disturbances already present with a subsequent heightened risk of falls [17,18,19,20,21]. Therefore, new therapies with minimal or no systemic side effects are needed.

Topical analgesics have a better safety profile compared to oral neuropathic pain medications, primarily due to their localized action, resulting in minimal to no systemic absorption and thus (nearly) no risk of systemic side effects. This localized delivery avoids the potential for interactions with oral medications, making them a safer option for patients with polypharmacy or those at risk of drug–drug interactions. Additionally, topical treatments may provide targeted pain relief with a faster onset of action and improved tolerability, particularly in patients with comorbidities that contraindicate systemic therapies [22].

Market-approved, topically applied analgesics are capsaicin 8% and lidocaine 5% patch [23]. However, since PDN primarily affects the toes and feet, handling of these patches can be rather inconvenient. Therefore, topical analgesic creams, such as phenytoin cream, are gaining interest due to their excellent safety profile and ease of application [24,25,26,27]. Until now, no systemic side effects or detectable phenytoin plasma levels have been reported after topical phenytoin cream application up to a concentration of 30% phenytoin in a cream [28]. However, studies on phenytoin cream encompassed many different etiologies of neuropathic pain, with their different pathophysiological mechanisms.

Prompted by the apparent swift onset of analgesic action (within 30 min), we developed various response tests from open to single-blind and ultimately double-blind tests that not only could easily discern direct responsiveness at the bedside but could also aid to appraise the likelihood of prolonged pain relief.

The objective of this study was to investigate the analgesic effect of topical phenytoin cream in patients with distal symmetrical sensorimotor type of PDN (i.e., polyneuropathy) and a positive response test. This exploration includes fast analgesic effects observed in response tests, the assessment of sustained analgesic effects following extended usage, and correlations between these effects.

## 2. Results

A response test with phenytoin cream was performed in 65 patients with PDN, including 16 participants from previous studies [25,26,27]. Demographic data, disease characteristics, and baseline pain scores are summarized in Table 1. A total of 31 patients (47.7%) demonstrated a positive response test. Specifically, 19/42 (45.2%) had a positive open response test (ORET), 4/6 (66.7%) had a positive single-blind placebo-controlled response test (SIBRET), and 8/17 (47.1%) had a positive double-blind placebo-controlled response test (DOBRET). Notably, 30 patients (42.2%) were already receiving neuropathic pain medications and still reported a median baseline pain score of 7.0 on the 11-point numerical rating scale (NRS) (IQR 6.0; 8.0). Neither local side effects, such as skin irritation or aggravation of pain, nor systemic side effects were reported in response tests or after the extended use of phenytoin cream.

### 2.1. Fast Analgesic Effect in Response Tests

Fast onset of pain relief was observed across all response tests, with a median pain reduction of 3.0 (IQR 2.0–4.0) NRS points compared to the baseline (*p* < 0.0001). No statistically significant difference in pain reduction was observed between patients who used neuropathic pain medication and those who did not.

In the SIBRET study, the application of phenytoin cream resulted in a statistically significant median difference in pain reduction compared with a placebo of 1.5 [IQR: 0.4–2.8] NRS points (*p* = 0.04).

The results of the DOBRET are presented in Table 2. A total of 12 patients underwent the DOBRET test with phenytoin 20% cream and 5 patients with phenytoin 10% cream. Overall, there was a mean difference in pain reduction between the phenytoin and placebo cream-applied areas of 1.8 (95% CI 0.8 to 2.8, *p* = 0.001) NRS points, corresponding to a mean difference in the percentage of pain reduction of 29.9% (95% CI 12.7% to 47.1%, *p* < 0.01). In patients with a positive DOBRET, these mean differences were 3.5 (95% CI 2.1 to 4.9, *p* = 0.0001) NRS points and 55.2% (95% CI 30.2% to 80.2%, *p* < 0.001)

### 2.2. Pain Relief After Extended Use

Table 3 provides detailed information on the effects of phenytoin cream after extended use. With a median duration of use of 3.3 (IQR 1.5–12.1) months, there was an extended median analgesic pain reduction of 4.0 (IQR 3.0–5.0) NRS points. A majority of patients experienced moderate (MPR30: *n* = 26; 83.9%) or considerable (MPR50; *n* = 21; 67.7%) pain relief.

Notably, one patient applied 10% phenytoin cream every 3 to 5 days and experienced complete relief from pins and needles within 1 min of application, with the effects lasting 3 to 5 days.

Patients who did not use additional neuropathic pain medication (*n* = 15) achieved an average pain reduction of 5 NRS points (IQR 4.0–5.5), compared to 3.0 NRS points (IQR 2.0–3.0) among those (*n* = 16) who did use additional pain medication (*p* = 0.007). However, when stratifying the analysis by response tests (ORET, SIBRET, and DOBRET), no statistically significant differences in pain relief were found between patients using additional neuropathic pain medication and those who did not.

### 2.3. Correlation Between Analgesic Effect in Positive Response Tests and After Extended Use

**Figure 1:**
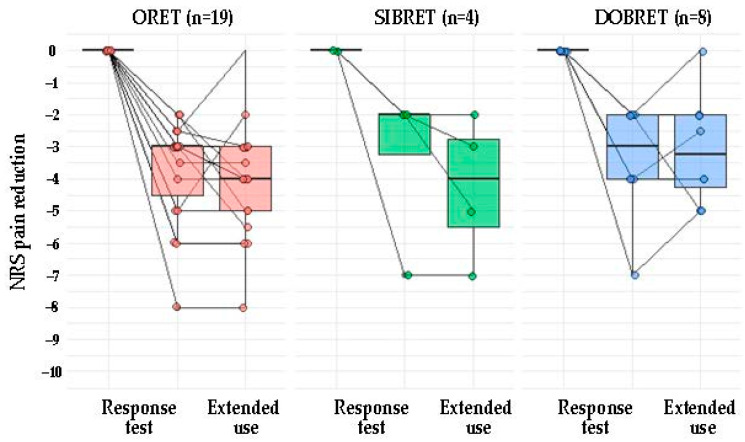
Pain reduction from baseline in PDN patients with positive response test: outcome of response tests and after extended topical phenytoin use. Note: Each dot represents an individual patient. The box plot illustrates the interquartile range, spanning from the 25th to the 75th percentile, with the line inside the box indicating the median. Abbreviations: PDN: painful diabetic neuropathy, DOBRET: double-blind, placebo-controlled response test, NRS: 11-point numerical rating scale, ORET: open response test, SIBRET: single-blind, placebo-controlled response test. To improve visualization, data points are jittered on the *x*-axis and *y*-axis to mitigate overlap.

**Figure 2:**
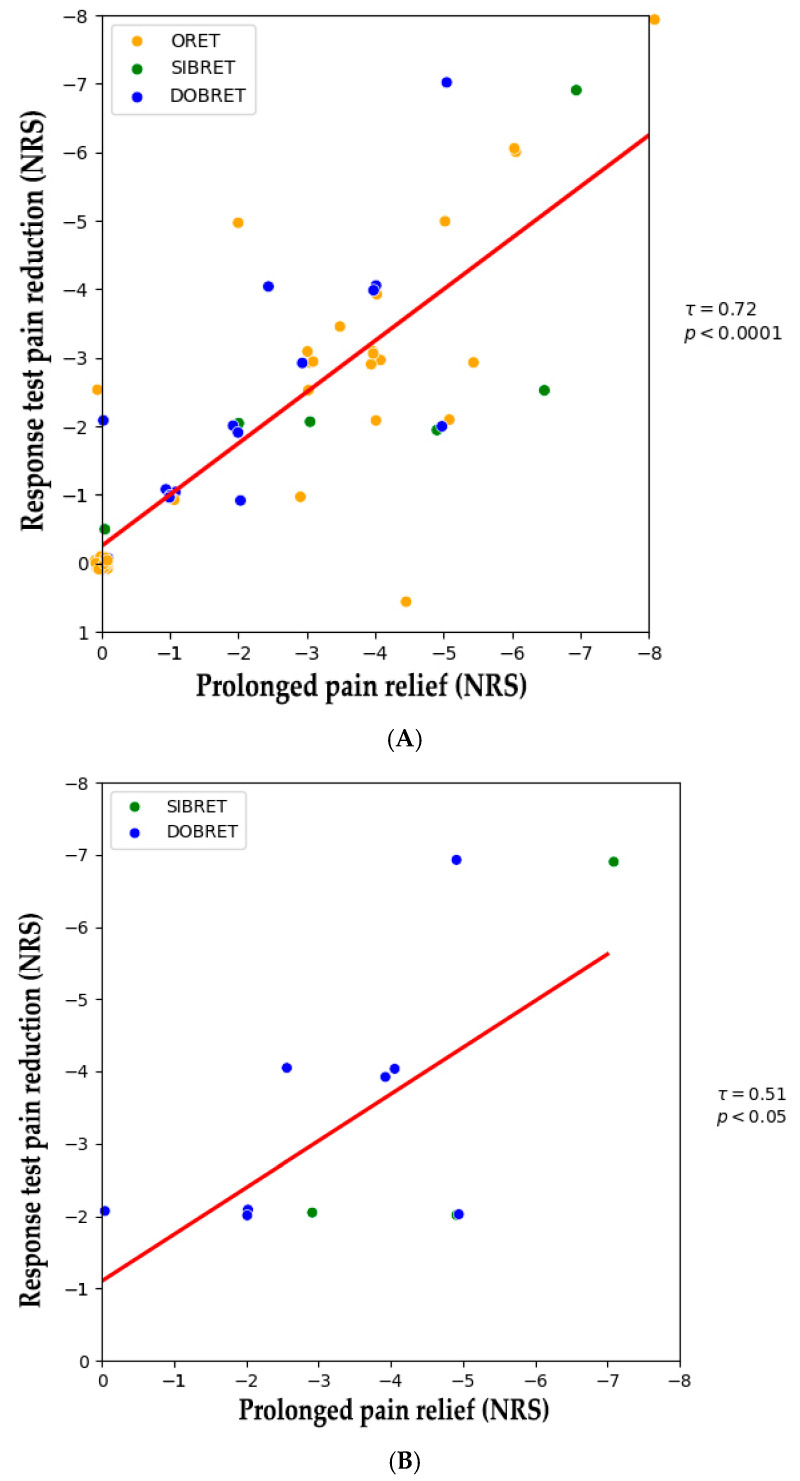
(**A**) Correlation between pain reduction in positive response tests and after extended topical phenytoin use. (**B**) Correlation between pain reduction in positive DOBRET and SIBRET and after extended topical phenytoin use. Note: Each dot represents an individual patient. The red line shows the linear regression fit, indicating a positive correlation between response test pain reduction and sustained pain relief. Abbreviations: DOBRET: double-blind, placebo-controlled response test, NRS: 11-point numerical rating scale, ORET: open response test, SIBRET: single-blind, placebo-controlled response test. To improve visualization, data points are jittered by ±0.1 on the *x*-axis and *y*-axis to mitigate overlap.

Figure 1 illustrates NRS changes in patients with positive response tests from the baseline to after the extended use of phenytoin cream. A positive correlation was observed between the analgesic effect during the response test and sustained pain relief following extended use (τ = 0.72, *p* < 0.0001), see Figure 2A. When considering patients with positive SIBRET or DOBRET separately (*n* = 12), a positive correlation was also found between the response test analgesic effect and sustained pain relief (τ = 0.51, *p* < 0.05), see Figure 2B. There were no significant correlations between sustained pain relief and other response test parameters, time to onset or duration of analgesia, or the applied quantity of the cream.

## 3. Discussion

Our study demonstrated a significant positive correlation between the fast analgesic effect of phenytoin cream observed in response tests and sustained pain relief after an average of 3 months use in PDN patients. This suggests that the response test can effectively identify patients likely to experience long-term neuropathic pain reduction with topical phenytoin treatment. Importantly, the time to onset of analgesic effect was not a prognostic factor. No studies have documented long-term effects in patients with a positive response test.

In line with prior studies, approximately 50% of patients had a positive response test in which a large majority of patients demonstrated sustained pain relief over time, and pain relief had a fast onset, was long lasting, and typically resulting in two daily applications [25,26,27]. We would also like to point out the additional pain reduction achieved in the majority of patients who were experiencing considerable baseline pain despite receiving systemic neuropathic pain medications.

The observed effectiveness of topical phenytoin in PDN is consistent with the findings from research on sensory nerve integrity. For example, a randomized trial with topical clonidine demonstrated a correlation between preserved sensory nerve function and pain reduction, suggesting that maintaining nerve integrity may be a critical factor for successful treatment outcomes [29]. This preservation could be tested with response tests.

Phenytoin exhibits a broad mechanism of actions by mainly inhibiting voltage-gated sodium (NaVs) and calcium channels (CaVs) in peripheral nerve endings and keratinocytes [30,31,32]. See Table 4 for main properties of phenytoin. Importantly, phenytoin cream demonstrates no systemic absorption when applied topically, highlighting its potential as an ideal candidate for localized applications [28]. This comprehensive channel blockade likely suppresses hyperexcitable nociceptors, contributing to pain reduction in patients with PDN.

Oral neuropathic pain medications have shown no significant differences in efficacy in head-to-head studies comparing tricyclic antidepressants (e.g., amitriptyline), pregabalin/gabapentin, serotonin–noradrenaline reuptake inhibitors (e.g., duloxetine), and opioids (e.g., morphine) [37]. While these medications remain widely used, their effectiveness varies, and side effects often limit their applicability.

Currently, market-approved topical analgesics for PDN treatment are limited to the capsaicin 8% patch, the only FDA-approved indication. Its approval is based on a double-blind, randomized controlled trial involving 369 PDN patients. This trial demonstrated superiority over placebo patches [38,39]. However, more than three-quarters of patients using the capsaicin 8% patch experienced side effects, primarily at the application site, with erythema being the most common, followed by pain [40].

Other topical treatments evaluated in randomized clinical trials, such as capsaicin 0.075%, amitriptyline 5%, clonidine 0.1%, ketamine 5%, lidocaine 5%, nutmeg extract, Citrullus colocynthis extract oil, glyceryl trinitrate 0.4%, and oxybutynin 3%, have shown inconsistent results or limited efficacy [18,41,42,43,44,45,46,47]. Direct comparisons between these treatments, as well as with phenytoin, are challenging due to variations in study designs, treatment phases, and the duration of trials. Future research is needed to establish the efficacy and comparative performance of these topical therapies.

The current research landscape highlights a significant gap in the development of novel topical analgesics for PDN. As of now, ClinicalTrials.gov lists no ongoing trials investigating new topical treatments, underscoring the urgent need for further research to identify more effective and tolerable options.

Our study has limitations. The predictive value of the response test remains uncertain as patients with a negative response test did not continue the phenytoin treatment. Also, the retrospective design and the small sample size limit our ability to draw firm conclusions, as both reduce control over confounding factors.

## 4. Materials and Methods

This study was reviewed and approved by the Institutional Review Board of Amsterdam UMC, location: Vrije Universiteit Amsterdam, the Netherlands (2022.0773). We retrospectively collected data from patients with PDN who visited the Institute for Neuropathic Pain between November 2016 and February 2023. The inclusion criteria included (1) diagnosis of PDN, defined as distal symmetrical sensory or sensorimotor polyneuropathy of the limbs and diabetes mellitus as the sole underlying cause or risk factor; (2) baseline pain intensity of at least 4 on the NRS; (3) scoring at least 4 on the DN4; (4) and tested with phenytoin cream 10% or 20%. The neuropathic pain characteristics of the DN4 include burning, painful cold, electric shocks, tingling, pins and needles, itch, numbness, hypesthesia for touch, and pinprick and allodynia [21]. Exclusion criteria included an inability to rate pain levels on the NRS and the presence of other known potential causes or risk factors for polyneuropathy. All patient data were handled with strict confidentiality.

### 4.1. Response Tests

Response tests were conducted when the pain intensity was ≥4 on the NRS and with the purpose to identify patients experiencing a fast predefined level of pain reduction within 30 min after cream application (i.e., a positive response test). Response tests were conducted only once in each patient. The creams consisted of a cetomacrogol base with or without phenytoin as the active pharmaceutical ingredient.

Three types of response tests were employed as follows:

(1) ORET: Phenytoin cream was applied to the most painful area, with both the patient and the treating physician unblinded. A positive ORET was defined as pain reduction of at least 2 NRS points [25]. To minimize the placebo effect, ORET was substituted with placebo-controlled tests whenever feasible and only performed in the clinic.
(2) SIBRET: In this test, phenytoin cream and a placebo cream were applied to two separate painful areas. Only the patient was blinded in this test [26]. Over time, to further reduce and prevent assessment bias, SIBRET was replaced by the double-blind testing method.
(3) DOBRET: A double-blinded version of SIBRET [27].

For both SIBRET and DOBRET, unblinding was done after the test.

Procedures to conduct SIBRET and DOBRET have been previously described in detail elsewhere [26,27]. Briefly, in both SIBRET and DOBRET, phenytoin cream and placebo cream were applied to two anatomically symmetrical painful areas (e.g., feet), with baseline pain intensity in both areas being similar or differing by no more than 1 point on the NRS. Pain intensity was recorded before and 30 min after cream application, and patients were monitored for potential side effects. A positive response was defined as pain reduction of at least 2 points on the NRS, with an additional minimum reduction of 1-point favoring the area treated with phenytoin cream.

Initially, 10% phenytoin cream was used in response tests; however, this was later replaced by 20% phenytoin cream, as it appeared to provide better pain relief, while maintaining good tolerability without causing side effects. The pharmacist compounded the phenytoin with cetomacrogol cream as excipient cream. Phenytoin creams and placebo creams were identical in appearance (white) and had the same feel. The placebo cream consisted of cetomacrogol cream without any active ingredient.

### 4.2. Extended Use and Pain Relief Monitoring

Patients who demonstrated a positive response to either 10% or 20% phenytoin cream during the response tests were prescribed that concentration. They were typically reassessed after 6 weeks of daily use, and subsequently every 2 months. At each follow-up, patients were asked to rate their average pain level over the previous week. The most recent pain score was used to calculate the degree of pain reduction to evaluate pain relief after extended use. Follow-up assessments focused on evaluating prolonged pain relief, onset of action post-application, duration of effect after a single application, frequency of daily applications, and the daily quantity of cream used. The quantity of cream applied was estimated with the finger-tip unit (FTU) that represents approximately 0.6 g of cream, sufficient to cover the distal phalanx of the index finger. Additional follow-up data were obtained during outpatient visits or through phone consultations when patients required a new yearly prescription. Patients lost to follow-up were included in the analysis using their most recent available data.

### 4.3. Statistical Analysis

Patient characteristics are summarized as the mean (standard deviation) for normally distributed data or as the median with interquartile range (IQR, 25th and 75th percentiles) for non-normally distributed data. Categorical data are reported as frequency (proportion). We compared differences in pain reduction between phenytoin and placebo creams, measured on the NRS, with the Wilcoxon signed-rank test. We conducted sub-analyses comparing pain reduction between neuropathic pain medication users and non-users using the Mann–Whitney U test. Additionally, to analyze the analgesic effect in patients with a positive DOBRET, we used a linear mixed-effects (LME) model incorporating a random intercept for each subject and a fixed effect for treatment (phenytoin or placebo), adjusted for baseline NRS scores (analysis of covariance) [27]. *p*-values in the LME analysis were derived from the likelihood ratio test; 95% confidence intervals (95% CI) around effect estimates were based on the profile likelihood.

We also determined the proportion of patients achieving minimum pain relief (MPR) on the NRS, defined as at least a 30% reduction from the baseline (MPR30, indicating moderate pain relief) and at least a 50% reduction (MPR50, indicating substantial pain relief) [48]. Associations between various response test parameters and sustained pain relief after the extended use of topical phenytoin cream were explored with Kendall’s tau correlation coefficient (two-tailed). All statistical analyses were conducted in SPSS, version 22 (SPSS Inc., Chicago, IL, USA) and statistical significance was set at *p*-values < 0.05.

## 5. Conclusions

Phenytoin cream shows potential as a safe and prolonged effective treatment for pain relief in PDN, particularly in patients identified with a positive response test. Future research should aim to include a broader patient population with neuropathic pain and explore long-term outcomes also in patients with a negative response test. Furthermore, prospective placebo-controlled phase III trials are warranted to confirm the efficacy of topical phenytoin cream and establish it as a standard treatment option for managing PDN and other neuropathic pain conditions.

## Figures and Tables

**Table 1:** Baseline patient characteristics.

	All Patients *n* = 65	ORET+ *n* = 19 of 42	SIBRET+ *n* = 4 of 6	DOBRET+ *n* = 8 of 17
Age, median (IQR)	66.0 (58.0–75.0)	69.0 (56.0–75.0)	67.5 (58.0–74.0)	75.0 (61.0–78.8)
Men/women	47/18	13/6	2/2	6/2
Years of pain, median (IQR)	4.0 (2.0–9.3)	5.0 (2.0–10.0)	6.0 (1.9–16.0)	2.8 (2.1–5.5)
Baseline NRS pain intensity, median (IQR)	7.0 (6.0–8.0)	8.0 (7.0 –8.0)	7.0 (5.5–7.8)	7.0 (5.5–8.0)
Diabetes Mellitus type 1/2, *n*/*n*	4/61	0/19	0/4	1/7
Use of neuropathic pain medication, *n* (%)	30 (46.2)	8 (42.1)	3 (75.0)	5 (62.5)
Use of regular pain medication, *n* (%)	12 (18.5)	6 (31.6)	0	1 (12.5)
**Anatomical Areas of Pain**	***n* (%)**	***n* (%)**	***n* (%)**	***n* (%)**
Toes only	4 (6.2)	1 (5.3)	0	1 (12.5)
Fore feet only	13 (20.0)	3 (15.8)	2 (50.0)	0
Soles only	2 (3.1)	0	0	0
Up to Ankles	24 (36.9)	10 (52.6)	0	1 (12.5)
Up to halfway lower legs	11 (16.9)	0	1 (25.0)	4 (50.0)
Up to knees	11 (16.9)	5 (26.3)	1 (25.0)	2 (25.0)
**Pain Characteristics**	***n* (%)**	***n* (%)**	***n* (%)**	***n* (%)**
Burning	50 (76.9)	16 (84.2)	3 (75.0)	5 (62.5)
Painful cold	23 (35.4)	9 (47.4)	0	2 (25.0)
Electric shocks	18 (27.7)	6 (31.6)	1 (25.0)	1 (12.5)
Tingling	54 (83.1)	17 (89.5)	4 (100)	7 (87.5)
Pins and needles	58 (89.2)	18 (94.7)	3 (75.0)	7 (87.5)
Itch	6 (9.2)	4 (21.1)	0	1 (12.5)
Allodynia	27 (41.5)	10 (52.6)	2 (50.0)	3 (37.5)

Abbreviations: DOBRET+: positive response on double-blind, placebo-controlled response test, IQR: interquartile range, *n*: number of patients, NRS: 11-point numerical rating scale, ORET+: positive response on the open response test, and SIBRET+: positive response on the single-blind, placebo-controlled response test.

**Table 2:** DOBRET: analgesic effect of phenytoin cream versus placebo cream in patients with painful diabetic neuropathy.

NRS Pain Reduction	Phenytoin		Placebo		Mean Difference		
	Mean	95% CI	Mean	95% CI	Mean	95% CI	*p*-Value
All patients (*n* = 17)	2.1	1.3 to 2.8	0.3	−0.5 to 1.0	1.8	0.8 to 2.8	0.001
Positive response * (*n* = 8)	3.4	2.4 to 4.4	−0.1	−1.1 to 0.9	3.5	2.1 to 4.9	0.0001
Negative response (*n* = 9)	0.9	0.09 to 1.7	0.6	−0.2 to 1.4	0.3	−0.4 to 1.0	0.4
**% Pain Reduction**	**Phenytoin**		**Placebo**		**Mean Difference**		
	**Mean**	**95% CI**	**Mean**	**95% CI**	**Mean**	**95% CI**	***p*-Value**
All patients (*n* = 17)	32.6	20.4 to 44.7	2.7	−9.5 to 14.8	29.9	12.7 to 47.1	<0.01
Positive response * (*n* = 8)	52.1	34.4 to 69.7	−3.1	−20.8 to 14.6	55.2	30.2 to 80.2	<0.001
Negative response (*n* = 9)	15.2	1.4 to 29.0	7.8	−6.0 to 21.6	7.4	−7.3 to 22.1	0.3

Note: * Positive response is defined as ≥2 points in pain reduction on the NRS as well as ≥1 point difference between the phenytoin cream and the placebo cream-applied area in favor of phenytoin cream. Abbreviations: CI: confidence interval, DOBRET: double-blind, placebo-controlled response test, *n*: number of patients, and NRS: 11-point numerical rating scale.

**Table 3:** Extended use and effect of phenytoin cream in PDN patients with a positive response test.

	All Response Tests+ *n* = 31 (47.7%)	ORET+ *n* = 19 (45.2%)	SIBRET+ *n* = 4 (66.7%)	DOBRET+ *n* = 8 (47.1%)
	Median (IQR)	Median (IQR)	Median (IQR)	Median (IQR)
Time to onset of effect (minutes)	15.0 (10.0–16.3)	15.0 (10.0–22.5)	10.0 (6.3–13.8)	12.5 (3.3–15.0)
Duration of effect (hours)	6.0 (3.5–8.0)	5.0 (3.3–10.0)	6.0 (5.5–7.0)	6.0 (3.0–40.0)
Number of daily applications	2.3 (2.0–3.0)	2.0 (2.0–3.4)	2.5 (2.3–2.8)	2.0 (0.6–3.5)
Grams per application	0.6 (0.6–1.2)	0.6 (0.6–1.2)	1.2 (0.9–1.2)	0.6 (0.6–0.9)
Grams per daily application	2.4 (1.7–3.2)	2.4 (0.9–3.6)	2.4 (2.1–2.7)	2.4 (2.1–2.4)
Duration of use (months)	3.3 (1.5–12.1)	3.0 (1.5–10.9)	5.8 (1.4–11.3)	6.3 (1.6–34.8)
Sustained pain reduction (NRS)	4.0 (3.0–5.0) *	4.0 (3.0–5.0) *	4.0 (2.3–6.5)	3.3 (2.0–4.8) °
MPR 30, *n* (%)	26 (83.9)	17 (89.5)	4 (100)	5 (62.5)
MPR 50, *n* (%)	21 (67.7)	16 (84.2)	2 (50)	3 (37.5)
Phenytoin 20% cream use	15 (48.4)	9 (47.4)	0	6 (75.0)
Phenytoin 10% cream use	16 (51.6)	10 (52.6)	4 (100)	2 (25.0)

Abbreviations: PDN: painful diabetic neuropathy, DOBRET+: positive response on double-blind, placebo-controlled response test, IQR: interquartile range, MPR: number of patients reaching a minimum pain relief of 30% and 50% from baseline, *n*: number of patients, NRS: 11-point numerical rating scale, ORET+: positive response on the open response test, Response test+: positive response on a response test, SIBRET+: positive response on the single-blind, placebo-controlled response test. * *p* < 0.0001 compared to the baseline with Wilcoxon signed-rank test; ° *p* < 0.05 compared to the baseline with Wilcoxon signed-rank test.

**Table 4:** Phenytoin: chemical, pharmacodynamic, and pharmacokinetic properties.

Synonym	Diphenylhydantoin
Chemical formula	C_15_H_12_N_2_O_2_
Chemical name	5,5-diphenylhydantoin, 5,5-diphenylimidazolidin-2,4-edione
Molecular weight	252.268
CAS number	57-41-0
Pubchem CID	1775
Melting range:	286–298 °C
pKa	8.3
IC_50_ NaV 1.1: 6.4 ± 1.8 µM (inactivated state) [33]	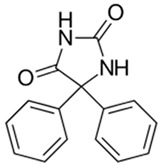
IC_50_ NaV 1.2: 11.8 ± 1.7 µM (inactivated state) [33]	
IC_50_ NaV 1.3: 7.1 µM (inactivated state) [33]	
IC_50_ NaV 1.5: 2.1 µM (inactivated state) [33]	
IC_50_ NaV 1.6: 7.7 ± 0.8 µM (inactivated state) [33]	
IC_50_ NaV 1.7: 4.0 ± 1.2 µM (inactivated state) [33]	
IC_50_ L-type CaV: 9.6 ± 2.1 µM [34]	
IC_50_ Calcium-impermeable AMPAR: 30 ± 4 µM [35]	
IC_50_ Calcium-permeable AMPAR: 250 ± 60 µM [35]	
Inducer of CYP2C9, CYP2C19, and CYP3A4 [36]	

IC_50_ Inhibitory concentration for 50% maximal effect.

## Data Availability

Deidentified participant data can be made available by request to the corresponding author. Requests will be considered after planned analyses and reporting have been completed by the investigators. Access will require submission of a protocol that is approved by a review committee and a signed data access agreement.
